# Validity of Perceived Stress Scale in Brazilian low-income college students

**DOI:** 10.11606/s1518-8787.2025059005974

**Published:** 2025-03-18

**Authors:** Ana Clara Arrais Rosa, Lorrane Cristine Conceição da Silva, Jacyara Christina Carvalho Azevedo, Rhavenna Thais Silva Oliveira, Ruhena Kelber Abrão Ferreira, Maíra Tristão Parra, Heráclito Barbosa Carvalho, Augusto César Ferreira de Moraes, Marcus Vinícius Nascimento-Ferreira

**Affiliations:** I Universidade Federal do Tocantins Programa de Pós-Graduação em Ensino em Ciência e Saúde Palmas TO Brasil Universidade Federal do Tocantins. Programa de Pós-Graduação em Ensino em Ciência e Saúde. Palmas, TO, Brasil; II Instituto de Ensino Superior do Sul do Maranhão Imperatriz MA Brasil Instituto de Ensino Superior do Sul do Maranhão. Imperatriz, MA, Brasil; III Universidade Federal do Tocantins Miracema do Tocantins TO Brasil Universidade Federal do Tocantins. Miracema do Tocantins, TO, Brasil; IV Universidade Estadual Paulista Faculdade de Medicina Botucatu SP Brasil Universidade Estadual Paulista. Faculdade de Medicina. Botucatu, SP, Brasil; V International Consulting Associates Arlington VA USA International Consulting Associates. Arlington, VA, USA; VI Universidade de São Paulo Faculdade de Medicina Departamento de Medicina Preventiva São Paulo SP Brasil Universidade de São Paulo. Faculdade de Medicina. Departamento de Medicina Preventiva. São Paulo, SP, Brasil; VII University of Texas Health Science Center at Houston School of Public Health Department of Epidemiology Austin TX USA University of Texas Health Science Center at Houston. School of Public Health. Department of Epidemiology. Austin, TX, USA

**Keywords:** Mental Health, Psychometrics, Adult, Income, Unsupervised Machine Learning

## Abstract

**OBJECTIVE:**

We tested the reliability and validity of the Perceived Stress Scale, an online questionnaire, among college students from low-income Brazilian regions.

**METHODS:**

We assessed 195 college students from a region with a Gini index of 0.56 for the validity study and a subsample of 117 students for the reliability study, where we evaluated the Perceived Stress Scale with the 14 original items. We also applied the shortened version of the Brief Symptom Inventory with 18 items (BSI-18). The psychometric properties analyzed, including temporal stability, internal consistency, and structural and convergent validity, were assessed using Spearman’s correlation coefficient, Cronbach’s alpha coefficient, unsupervised machine learning, and confirmatory factor analysis.

**RESULTS:**

The questionnaire showed acceptable reliability (temporal stability [rho ≥ 0.32] and internal consistency [alpha ≥ 0.79]). In construct validity, we identified two clusters, “helplessness” and “self-efficacy”, as structure solutions for our sample via unsupervised machine learning. An acceptable fit for the two-factor structure of the scale was indicated by multiple indices (chi-square/degrees of freedom [χ2/df] = 119/76; Tucker-Lewis Index [TLI] = 0.916; Comparative Fit Index [CFI] = 0.930; root mean square error of approximation [RMSEA] = 0.054; standardized root mean-squared residual [SRMR] = 0.078)) on confirmatory factor analysis. Moreover, convergent validity was supported by significant correlations of the BSI-18 Global Severity Index score with perception of helplessness (rho = 0.71) and self-efficacy (rho = -0.42).

**CONCLUSION:**

The Perceived Stress Scale, which is an online tool, is a reliable and valid self-report tool for college students.

## INTRODUCTION

The Perceived Stress Scale is one of the most commonly used tools to measure stress globally^1^. This questionnaire was initially developed for college students^2^ and evaluates the degree to which individuals believe their life has been unpredictable, uncontrollable, or overloading during the previous month^1^. The assessed items are general rather than specific events or experiences^1^. The three versions (14, 10 or four items) of this subjective tool have low economic and logistical costs and possess psychometric properties acceptable to several countries^1,3-7^. To infer stress levels, the scale evaluates two dimensions (negative [representing the perception of helplessness] and positive [representing the perception of self-efficacy]) of perceived stress^6^. Scale validity is not, however, an obvious or straightforward endeavor.

In general, the development and validation of a subjective tool (e.g., scale, questionnaire, log, diary) are conducted simultaneously, but additional psychometric assessments are needed to apply this tool to another population^8^. Without demonstrating measurement validity, it is unknown whether observed differences in scores across respondents reflect true differences in stress or differences in how these groups define, experience, and communicate stress^9^. In the case of the Perceived Stress Scale, based on 14 items, a comprehensive systematic literature review indicated that one- and two-factor were possible structure solutions^1^ The latter structure solution was reported as consensus in the literature^10^. An online psychometric evaluation during the COVID-19 pandemic also identified two-factor solutions in the Chinese adult population^11^. On the other hand, another recent psychometric evaluation with a representative sample in Australia observed item misfit (the fit of the one-factor model was rejected), suggesting item exclusion and encouraging the development of new items to ensure construct representation^10^.

In Brazil, there is no consensus in the literature regarding the dimensionality of the Perceived Stress Scale^5^. The initial study on the psychometric properties of the original 14-item version was conducted in an elderly population^4^, with the translation process involved translation, back-translation, and committee review. This study demonstrated that the scale is a clear and reliable tool for measuring perceived stress in this demographic^4^. However, subsequent studies using this translated version have shown inconclusive results regarding its dimensionality, with both one- and two-factor structures identified in samples of female college students^12^ and pregnant women^5^, respectively.

The dimensionality equivalence in Brazilian college students (or at least in adults) has not yet been established, while the test-retest reliability and criterion validity have been relatively rarely evaluated across different populations and contexts^1^. Additionally, there is limited literature on the psychometric properties of the online version of the Perceived Stress Scale. Although the adoption of online tools has been increased during the COVID-19^13^, the level of education of the participants and access to the internet may distort recruitment and the ability to respond to online tools, mainly in populations with marked socioeconomic and educational differences^13^. Therefore, we aimed to test the reliability (temporal stability and internal consistency) and validity (structural and convergent) of the online Perceived Stress Scale for college students in low-income Brazilian regions.

## METHODS

### Study Design

This is a methodological study carried out in the first stage of a longitudinal observational multicenter project entitled 24-hour movement behavior and metabolic syndrome (24h-MESYN). A detailed description of the 24h-MESYN study can be found elsewhere^RR^.^14^ In this phase, participants answered the same questionnaire twice, with an interval of two weeks, to verify the reliability (temporal stability and internal consistency of the responses) and the (structural and convergent) validity of the construct. Data collection occurred during the first semester of 2021.

### Ethical Aspects

This project was approved by the Research Ethics Committee of *Universidade Federal do Tocantins* (No 5,161,340) and *Centro Universitário do Maranhão* (UNICEUMA, No 4,055,604). The project follows the provisions of the Declaration of Helsinki, revised in 2008, in Seoul, Korea; the resolution of CNS 466/12; the guidelines for conducting research activity during the COVID-19 pandemic (available at: http://www.fo.usp.br/wp-content/uploads/2020/07/Orientações-condução-de-pesquisas-e-atividades-CEP.pdf) and the guidelines for research in a virtual environment (OFICIO CIRCULAR No. 2/2021/CONEP/SECNS/MS). Students interested in participating in the study were invited to sign an online informed consent form.

### Population, Sample and Sampling

The study population consisted of students enrolled in a higher education institution in the city of Imperatriz, in the state of Maranhão (located in the Northeast of Brazil), selected by convenience. In Imperatriz, the most recent data regarding income distribution showed a *Gini* index of 0.56 (1.00 implies perfect inequality) in 2010^15^, whereas Maranhão state has an index of 0.53, which was most recently measured in 2022^15^. In 2020, the institution had 2,225 students enrolled in nine undergraduate programs (Administration, Law, Physical Education, Nursing, Esthetics and Cosmetics, Physiotherapy, Nutrition, Psychology and Social Work)^RR^. The parameters used to calculate the sample size were α of 0.05, β of 0.10 (or power of 90%), and Pearson’s correlation coefficient = 0.30 (minimum required for a correlation matrix in an exploratory factor analysis)^16^. Based on these parameters, a sample of 85 individuals was estimated. Predicting losses and rejections of 50% in the first and second application^18^, we established a minimum sample of 170 participants to study the psychometric properties of the Perceived Stress Scale. However, the 24h-MESYN study was designed to evaluate the psychometric properties of other subjective tools, such as the International Physical Activity Questionnaire (IPAQ)^RR^, for example. Based on estimates of less robust psychometric properties, we designed a sample for the project with 342 invited participants, aiming to meet minimum aspects (α of 0.05 and β of 0.10) for all tested tools. A stratified random sampling of students was used based on a previous representative cohort according to sex (at least 60.0% for females), age (at least 25.0% for students up to 20 years of age), and study program (at least 60.0% in the health area)^18^. The potential participants were sampled in the college entrance hall and open areas in accordance with health guidelines.

### Inclusion and Exclusion Criteria

All regularly enrolled students aged at least 17 years who were selected for the study and signed an informed consent form were enrolled. The exclusion criteria were pregnancy, physical disability or not completing the questionnaires. In the case of pregnancy and physical disability, students were evaluated but were excluded from the analyses.

### Study Variables and Instruments

The study variables were sex, age, academic shift and course, perceived stress, and psychological distress. The information was accessed via an online form (available at https://forms.gle/L92wXsVaxxfPNgpE8, accessed on november 29 2024). We evaluated the responses using self-reported questionnaires in Portuguese as follows:

•Demographics and academic characteristics: sex, age, academic shift, and course;

•Perceived stress: We adopted the Portuguese version of the Perceived Stress Scale^4^. This scale, in its original version with 14 items^2^, has been validated in Brazil for female college students^12^ and pregnant women^5^. The semantic, idiomatic, experimental, cultural, and conceptual equivalences were previously evaluated^4^. The instrument is self-reported and measures perceived stress via (items) feelings and thoughts during the last month (Table 1). On the original scale, seven are considered negative dimension (helplessness; Items 1, 2, 3, 8, 11, 12 and 14), and seven were considered positive dimension (self-efficacy; Items 4, 5, 6, 7, 9, 10 and 13)^2^. The responses were measured on a five-point Likert scale (0 = never to 4 = very often) on the frequency of feeling/thought^6^. On the negative dimension subscale, the score was based on the sum of the points for each item^6^. On the positive dimension subscale, the score was calculated after inverting the scores of the items and then adding them together^6^. The total score ranged from 0 to 56 points^6^. Higher scores indicate greater perceived stress^6^.

**Table 1:** Perceived Stress Scale with 14 items in Portuguese^4^.

Neste último mês, com que frequencia...
1 Você tem ficado triste por causa de algo que aconteceu inesperadamente?
2 Você tem se sentido incapaz de controlar as coisas importantes em sua vida?
3 Você tem se sentido nervoso e “estressado”?
4 Você tem tratado com sucesso dos problemas difíceis da vida?
5 Você tem sentido que está lidando bem as mudanças importantes que estão ocorrendo em sua vida?
6 Você tem se sentido confiante na sua habilidade de resolver problemas pessoais?
7 Você tem sentido que as coisas estão acontecendo de acordo com a sua vontade?
8 Você tem achado que não conseguiria lidar com todas as coisas que você tem que fazer?
9 Você tem conseguido controlar as irritações em sua vida?
10 Você tem sentido que as coisas estão sob o seu controle?
11 Você tem ficado irritado porque as coisas que acontecem estão fora do seu controle?
12 Você tem se encontrado pensando sobre as coisas que deve fazer?
13 Você tem conseguido controlar a maneira como gasta seu tempo?
14 Você tem sentido que as dificuldades se acumulam a ponto de você acreditar que não pode superá-las?

•Psychological distress: The Brief Symptom Inventory with 18 items (BSI-18)^19^ was validated to assess psychological distress in the general population^20^, including Brazilian adults^23^. The questionnaire required the participants to evaluate the extent of distress or annoyance they had experienced^20^. Responses were rated on a five-point Likert scale, ranging from 0 (not at all) to 4 (very much). Higher scores indicate greater psychological distress^21^. In our sample, the BSI-18 showed acceptable internal consistency (Cronbach’s alpha coefficient = 0.92, 0.78, 0.89, and 0.86 for Global Severity Index, Somatization, Depression, and Anxiety, respectively).

### Procedures

We conducted the research in three stages. In the first stage, we invited students to participate in the study in person, observing all sanitary and institutional standards related to the pandemic. In this step, we explained the project and sent the link with an informed consent form via an instant messaging application (WhatsApp). In the second stage, after obtaining an electronic signature on the consent form, the participants answered the questionnaire (Q1, first application). In the third stage, two weeks after the first stage, we send a reminder of the link, and the participants answer the same questionnaire again (Q2, second application). The questionnaire was sent to only those who replied to the questionnaire in Q1.

### Statistical Analysis

We performed all statistical analyses using Stata software (version 15.0, Stata Corporation, College Station, USA) and a p ≤ 0.05 was considered significant. In the sensitivity analysis, we applied the chi-square goodness-of-fit test, considering Q1 as the observed proportion and Q2 as the expected proportion. In the reliability analysis, we applied Spearman’s correlation coefficient (rho; Q1 vs Q2) with a cutoff of ≥ 0.30 to indicate acceptable temporal stability^22^ and Cronbach’s alpha coefficient (alpha; Q1 only) with a cutoff of ≥ 0.70 to indicate internal consistency reliability ^23^.

In the construct (structural) validity analysis, we first conducted unsupervised machine learning (to identify the construct structure solution of our dataset). This procedure assesses the extent to which an instrument reveals its internal structure as expected or theoretically hypothesized^24^. Thus, we adopted this dimension reduction technique^25^ to find undefined patterns or clusters^26^ that can be comparable to dimensions (from original Perceived Stress Scale, for example). Initially, we graphically explored potential clusters graphically with a (dendrogram) hierarchical method using complete linkage (maximum distance from the furthest neighbor) cluster analysis^28^. Sequentially, we adopted the Calinski–Harabasz index to determine the number of clusters, where the largest value indicates distinct clustering^31^. After identifying the clusters, we applied the k-mean method to create the clusters^31^, the most frequently used unsupervised learning classifier^29^. We used Student’s t-test for unpaired samples to compare the scores reported in the original Perceived Stress Scale stratified by machine learning clusters.

Second, we conducted a confirmatory factor analysis (to test if the two-factor structure solution [original theoretical model^2,5^] fits in our dataset). We employed structural equation modeling with a maximum likelihood estimator^17^. The measured items (item 1 to item 14) were included as endogenous variables, whereas the latent domains (helplessness and self-efficacy) were treated as exogenous variables^17^. We evaluated model quality based on the following parameters: Tucker–Lewis Index (TLI > 0.90), Comparative Fit Index (CFI > 0.90), root mean square error of approximation (RMSEA < 0.06), and standardized root mean square residual (SRMR < 0.08)^30^.

In the convergent validity analysis, we assessed the correlation between Perceived Stress and BSI-18. We evaluated via bivariate Spearman correlation coefficients the convergence between scores of helplessness and self-efficacy with the Global Severity Index (GSI), including all 18 items. A cutoff ≥ 0.30 was considered acceptable^24^ convergence validity.

## RESULTS

In Q1, 195 students answered the questionnaire, while in Q2, 117 answered it. Most of the students were female (74.9%, Q1; 72.6%, Q2), many were between 21 and 25 years old (44.6%, Q1; 45.7%, Q2), most were attending school at night (61.3%, Q1; 62.4%, Q2) and some were attending physical education (24.0%, Q1; 24.8%, Q2). In the sensitivity analysis, no significant differences were found (p > 0.05); therefore, there was no differential bias between the responses of Q1 and Q2. However, 43.0% of the patients refused to participate in the study in Q1, and a cumulative proportion of 65.8% refused to participate in Q2 (Table 2).

**Table 2:** Description of the sample and sensitivity analysis according to demographic and academic variables in the first and second questionnaire application.

Variables	Q1	Q2	p-value^a^
	(n = 195)	(n = 117)	
	n (%)	n (%)	
Biological sex			
Male	49 (25.1)	32 (27.4)	0.36
Female	146 (74.9)	85 (72.6)	
Age (years)			
≤ 20	46 (23.6)	31 (26.7)	0.63
21–25	87 (44.6)	53 (45.7)	
26–30	36 (18.5)	17 (14.7)	
31–35	14 (7.2)	6 (5.2)	
≥ 36	12 (6.2)	9 (7.8)	
Academic shift			0.92
Morning	39 (20.1)	24 (20.5)	
Evening	38 (18.6)	20 (17.1)	
Night	119 (61.3)	73 (62.4)	
Academic course			
Nutrition	15 (7.7)	7 (6.0)	0.17
Physical Education	47 (24.0)	29 (24.8)	
Nursing	25 (12.8)	14 (12.0)	
Esthetics and Cosmetics	8 (4.1)	2 (1.7)	
Physiotherapy	34 (17.3)	22 (18.8)	
Law	19 (9.7)	13 (11.1)	
Psychology	39 (19.9)	25 (21.4)	
Social work	5 (2.6)	3 (2.6)	

Q1: first questionnaire application; Q2: second questionnaire application.

^a^ Chi-square goodness-of-fit test.

Table 3 presents the reliability analysis. The questionnaire showed acceptable temporal stability, with a Spearman correlation coefficient between 0.32 (Item 8) and 0.65 (Item 3). Additionally, an acceptable correlation was observed for the negative domain (rho = 0.74), positive domain (rho = 0.64), and full scale (rho = 0.75) of the original version of the Perceived Stress Scale. The full scale also demonstrated acceptable internal consistency, with a Cronbach’s alpha of 0.84 (0.85 and 0.84 for the negative and positive domains, respectively).

**Table 3:** Reliability analysis of the Perceived Stress Scale.

Items	Q1	Q2	rho	alpha
	Median (p25–p75)	Median (p25–p75)		
Item 1	2.0 (1.0–3.0)	2.0 (1.0–3.0)	**0.49**	0.82
Item 2	2.0 (1.0–3.0)	2.0 (1.0–3.0)	**0.57**	0.80
Item 3	2.0 (2.0–3.0)	2.0 (2.0–3.0)	**0.65**	0.81
Item 8	2.0 (2.0–3.0)	2.0 (2.0–3.0)	**0.32**	0.85
Item 11	2.0 (1.0–3.0)	2.0 (2.0–2.0)	**0.46**	0.82
Item 12	2.0 (2.0–3.0)	2.0 (2.0–3.0)	**0.47**	0.86
Item 14	2.0 (1.0–2.0)	2.0 (1.0–2.0)	**0.49**	0.82
Negative domain^a^	15.0 (12.0–19.0)	14.0 (12.0–17.0)	**0.74**	0.85
Item 4	2.0 (2.0–3.0)	2.0 (1.0–2.0)	**0.47**	0.81
Item 5	2.0 (2.0–3.0)	2.0 (1.0–2.0)	**0.46**	0.80
Item 6	2.0 (2.0–3.0)	2.0 (1.0–2.0)	**0.48**	0.79
Item 7	2.0 (1.0–2.0)	2.0 (1.0–2.0)	**0.41**	0.82
Item 9	2.0 (1.0–2.0)	2.0 (1.0–2.0)	**0.57**	0.82
Item 10	2.0 (1.0–2.0)	2.0 (1.0–2.0)	**0.37**	0.82
Item 13	2.0 (1.0–2.0)	2.0 (1.0–2.0)	**0.56**	0.83
Positive domain^b^	13.0 (11.0–16.0)	13.0 (9.0–15.0)	**0.64**	0.84
Full scale ^c^	29.0 (24.0–35.0)	29.0 (26.0–36.0)	**0.75**	0.84

Significant values are in **bold (p < 0.05)**. alpha, Cronbach’s alpha coefficient; p25, 25th percentile; p75, 75th percentile; Q1, first application of the questionnaire; Q2, second application of the questionnaire.

^a^Based on the seven negative items in the original Perceived Stress Scale^4^.

^b^Based on the seven positive items in the original Perceived Stress Scale^4^.

^c^ Based on 14 items from the original Perceived Stress Scale^4^.

We identified a two-factor model as the structure solution via unsupervised machine learning assessment. In comparison with the one-cluster model, the two- and three-cluster showed Calinski/Harabasz index of 58.1 and 40.1, respectively. The dendrogram also showed the better fit for the two-factor model graphically (Figure A). In this sense, we extracted two clusters (factors) and labeled the machine learning clusters as “helplessness” (average points based on original Perceived Stress Scale: factor 1, mean of 19.1 [SD: 3.5]; and factor 2: mean of 11.2 [SD: 4.0]) and “self-efficacy” (average points based on original Perceived Stress Scale: factor 1, mean of 11.4 [SD: 4.0]; and factor 2: mean of 15.4 [SD: 3.9]) (Figure B). These clusters were able to identify differences in the negative (helplessness vs self-efficacy, p < 0.001) and positive (helplessness vs self-efficacy, p < 0.001) dimensions based on mean comparisons (Figure C).

**Figure:**
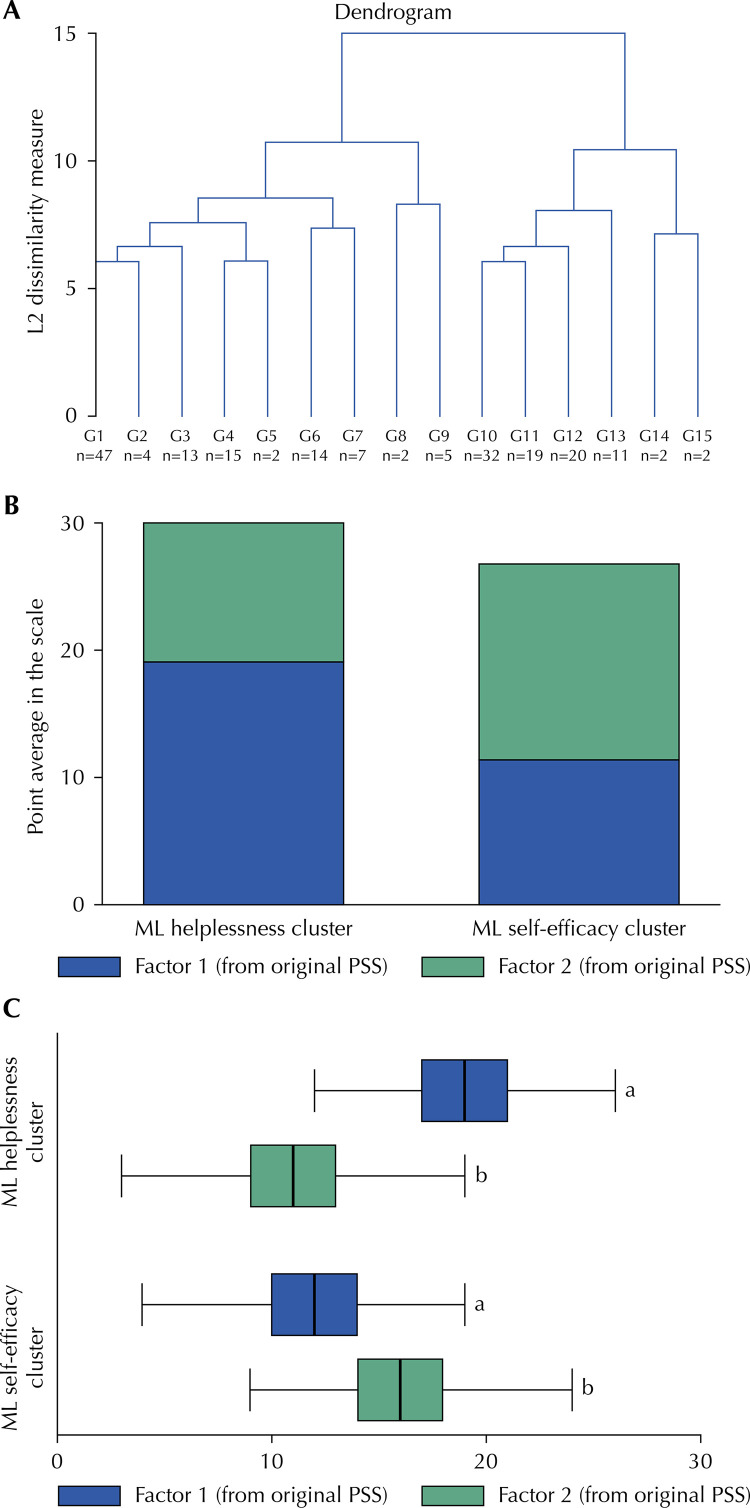
Validity analysis of the Perceived Stress Scale. Hierarchical cluster analysis (A), k-mean cluster group distribution (A), and mean differences (C).

ML: machine learning; PSS: Perceived Stress Scale.

^a^ Student’s t-test, p < 0.001.

^b^ Student’s t-test, p < 0.001.

Table 4 also presents the construct (structural) validity analysis. Here, we observed that the Perceived Stress Scale with two domains (helplessness and self-efficacy) had acceptable fitting in our sample, as originally planned in its theoretical model. Factor loadings ranged from 0.33 (item 8) to 0.94 (item 2) with a TLI of 0.916, CFI of 0.930, RMSEA of 0.353, and SRMR of 0.078. In the convergent validity analysis, we observed significant correlations between scores of helplessness (rho = 0.71; p < 0.001), self-efficacy (rho = -0.42; p < 0.001) and full scale (rho = 0.69; p < 0.001) with the BSI-18 Global Severity Index score.

**Table 4:** Validity analysis (confirmatory factor analysis) of the Perceived Stress Scale.

Structural equation modeling	Factor 1^a^		Factor 2^b^		β^c^	p-value	
	Loading	SE	Loading	SE			
Item 1	0.87	0.06			0.79	< 0.001	
Item 2	0.94	0.06			0.84	< 0.001	
Item 3	0.82	0.07			0.73	< 0.001	
Item 4			0.55	0.05	0.65	< 0.001	
Item 5			0.61	0.05	0.72	< 0.001	
Item 6			0.76	0.05	0.79	< 0.001	
Item 7			0.60	0.06	0.49	< 0.001	
Item 8	0.33	0.06			0.41	< 0.001	
Item 9			0.59	0.06	0.85	< 0.001	
Item 10			0.55	0.06	0.56	< 0.001	
Item 11	0.72	0.07			0.71	< 0.001	
Item 12	0.36	0.06			0.36	< 0.001	
Item 13			0.45	0.06	0.52	< 0.001	
Item 14	0.77	0.06			0.72	< 0.001	
**Fit indices**	**n**	**X^2^**	**df**	**TLI**	**CFI**	**RMSEA**	**SRMR**
Two-factor model	195	119	76	0.916	0.930	0.054	0.078
		(0.001)^d^				(0.353)^d^	

CFI, comparative fit index; df, degrees of freedom; n, number of observations; p, p-value; RMSEA, root mean square error of approximation; SRMR, standardized root mean square residual; SE, standard error; TLI, Tucker–Lewis Index; X^2^, chi-square.

^a^ Negative domain.

^b^ Positive domain.

^c^ Coefficient estimated by domain

^d^ p-value.

## DISCUSSION

This study assessed the reliability, construct, and convergent validity of the online version of the Perceived Stress Scale among college students from low-income Brazilian regions. The two-factor structure solution addressing helplessness and self-efficacy items with a single underlying general factor of perceived stress provided adequate fit for low-income college students and converged with psychological distress. Thus, the Perceived Stress Scale is a promising low-cost public health surveillance tool for monitoring stress during a pandemic, including social isolation, or even in remote-format research.

The temporal stability over a 2-week interval and the internal consistency of the Perceived Stress Scale were acceptable, according to our results. These findings are in line with previous studies that observed stability and consistency in versions with 14^1^ or 10 items^1,3^. A potential explanation can be attributed to the robust reliability of this tool for a period of two days to four weeks, with its performance diminishing after six weeks^1^. This reduced performance may imply that the duration of stability of the Perceived Stress Scale may be less than six weeks^1^ or that perceptions of life unpredictability, lack of control, and overload based on specific events or experiences may change within this period^1^. Additionally, we speculate that the use of short statements (only Item 14 is based on negation) (with up to 18 words, Item 5) may corroborate the stability of the questionnaire, whereas internal consistency tends to increase with the number of items in an instrument^1^.

In our study, construct validity was investigated based on structural assessment^17^, showing two factors as the structure solution, in line with the literature for the Perceived Stress Scale^10^, even in online assessments during the COVID-19 pandemic^11^. Corroborating our observed results indirectly, a study on Brazilian female college students demonstrated inadequate model fitting for a one-factor structure^12^. In addition to construct validity, this tool demonstrated acceptable convergence with psychological distress. This result is consistent with those of previous studies^9,31^. This ability can be partially explained by the theoretically related concepts of perceived stress and psychological distress, which are defined as either the stimulus or triggering event (the stressor) or the response or reaction (organic reaction generated by the stressor)^31^. This indicates that both instruments measure stress and distress constructs, respectively.

Using an unsupervised machine learning technique, our findings provide evidence to support a two-factor solution to the scale. Therefore, the Perceived Stress Scale in this sample indicates a sufficient capability for assessing differences between helplessness and self-efficacy. Moreover, based on the confirmatory analysis parameters, we obtain no evidence to reject the hypothesis of the two-factor model for college students from low-income regions as a structural solution. In this sense, our findings indicate that both data-driven [unsupervised machine learning] and theory-driven [confirmatory factor analysis], have two factors as structure solutions. Our study reflects the structure solution most reported in the literature^1^ and suggested as consensus as well^10^. We can potentially account for these findings by considering that different populations shared a perception of perceived stress, as evaluated using the Perceived Stress Scale.

Some limitations of this study should be mentioned. Although our sample was based on diverse parameters (age range, biological sex and nature of the course) found in a previous study^20^, generalization to the general population is not justified beyond psychometric findings. Thus, we believe that this sample would not differ from a random sample (from the same population) because its psychometric properties remained stable and valid in line with the literature^1^. Considering the lowest correlation observed (0.32; Item 8; Table 3), we observed a sample power of 99.0% (β = 0.01, one-tailed) in the post hoc analysis. Additionally, our sample size was sufficient to infer structural validity^17^ because we recruited the minimum required sample size of 10 participants per item for factor analysis^8,18^. Finally, the research site was selected for convenience, and the sampling process was random because the economic and logistical cost of a representative sample makes a methodological study unfeasible.

Our study also has several strengths. We contribute to stress measurement by investigating the Perceived Stress Scale within a stressful environment (college) and context (economically disadvantaged areas). Additionally, this study advances the literature by employing a machine learning technique to investigate psychometric properties for the first time. By compressing the information in a dataset into fewer factors or dimensions, issues such as multicollinearity or high computational costs can be avoided^27^ However, note that this procedure has limitations when comparing the fit of the target model to the observed data. The results of our study may motivate researchers to propose unsupervised learning as a dimension reduction technique^27^ for improved stress psychometric assessment.

## CONCLUSION

The online version of the Perceived Stress Scale has acceptable reliability and validity among Brazilian college students, and its application in health research and practice during a pandemic period is a low-cost alternative for monitoring stress in low-income regions.
